# Behavior of the Biological Control Agent *Bacillus thuringiensis* subsp. *aizawai* ABTS-1857 and *Salmonella enterica* on Spinach Plants and Cut Leaves

**DOI:** 10.3389/fmicb.2021.626029

**Published:** 2021-02-03

**Authors:** Xingchen Zhao, Marcelo Belchior Rosendo da Silva, Inge Van der Linden, Bernadette D. G. M. Franco, Mieke Uyttendaele

**Affiliations:** ^1^Food Microbiology and Food Preservation Research Unit, Department of Food Technology, Safety and Health, Faculty of Bioscience Engineering, Ghent University, Ghent, Belgium; ^2^FoRC – Food Research Center, Department of Food and Experimental Nutrition, Faculty of Pharmaceutical Sciences, University of São Paulo, São Paulo, Brazil

**Keywords:** biocontrol, XenTari^®^, *Bacillus cereus sensu lato*, *Salmonella*, spinach, pre-harvest, post-harvest

## Abstract

Fresh produce has been identified as an important vehicle for the transmission of foodborne pathogens. This study evaluated the behavior of vegetative cells and spores of *Bacillus thuringiensis*, one of the main biological control agents (BCAs) used in the world, and *Salmonella enterica* on spinach plants (pre-harvest) and spinach cut leaves (post-harvest) at 12°C, experimentally inoculated as single or co-cultures. The results evidenced that spray-inoculated commercial BCA containing *Bacillus thuringiensis* subsp. *aizawai* ABTS-1857 (BTa ABTS-1857) spores persisted well on spinach leaves in both pre- and post-harvest simulations. However, when BTa ABTS-1857 vegetative cells were spray-inoculated, more than 2 log reductions in the counts of *B. thuringiensis* were observed during 20 days pre- and 5 days post-harvest simulations, respectively. The counts of *S.* Montevideo on the spinach cut leaves during post-harvest storage at 12°C for 5 days remained unchanged, whereas 1 log reduction was noted during pre-harvest. Moreover, during pre-harvest simulation, when co-inoculated with BTa ABTS-1857 vegetative cells or spores, additional 0.5 or 1.0 log reductions were detected on the counts of *S.* Montevideo in the spinach leaves on the 10th day. These results were obtained under laboratory conditions, and further findings in longitudinal studies from farm (in the agricultural field) to retail (end of shelf life) will contribute to understanding of the role of *B. thuringiensis* as a BCA on growth/survival of *Salmonella* spp. in fresh produce.

## Introduction

Spinach (*Spinacia oleracea*) and other leafy greens are an important part of the healthy diet for humans being a rich source of vitamins and fiber. However, outbreaks associated with the consumption of fresh produce contaminated with pathogenic bacteria have increased in recent decades (Alegbeleye et al., 2018; Iwu and Okoh, 2019). In the farm to fork path of leafy greens, there are multiple opportunities for contact with human pathogens, that may happen during pre-harvest (contaminated manure, soil, irrigation water, livestock/wildlife, etc.), at harvest (leaves and stalks cutting off, trimming, washing, packaging, and transport) or post-harvest (cross-contamination) (Pérez-Rodríguez et al., 2011; Olaimat and Holley, 2012). The most commonly implicated enteric pathogens in leafy greens are Shiga toxin-producing *Escherichia coli* and *Salmonella* spp. (Schikora et al., 2012; Ceuppens et al., 2015; Uyttendaele et al., 2015; Centers for Disease Control and Prevention (CDC), 2020). In the EU and other countries, the main vehicles of foodborne diseases from non-animal origin were raw leafy greens, where *Salmonella* spp. was the most frequent infectious agent (Da Silva Felício et al., 2015; Fröhling et al., 2018).

Pesticides are often used in conventional agricultural production to minimize losses due to micro-organisms causing plant diseases or insects feeding on the plant. In organic farming, the use of synthetic chemical pesticides is restricted. One alternative for pests control is the use of plant protection products (PPP), comprised of living organisms called biological control agents (BCAs) (Sundh and Goettel, 2013). *Bacillus thuringiensis* is a soil-dwelling and gram-positive sporulating bacterium which belongs to *Bacillus cereus sensu lato*, also known as the *B. cereus* group. The *B. cereus* group comprises 18 genetically closely related species, with *Bacillus anthracis, B. cereus sensu stricto, B. thuringiensis, Bacillus mycoides, Bacillus pseudomycoides, Bacillus weihenstephanensis, Bacillus cytotoxicus*, and *Bacillus toyonensis* as the most prominent members (Guinebretière et al., 2008; Liu et al., 2015, 2017).

*Bacillus thuringiensis* used as PPP is the most successful and best-known BCA in the world, used for decades for the control of forest and agricultural pests. The insecticidal activity of *B. thuringiensis* is devoted to the production of multiple crystal proteins (δ-endotoxins) (Van Frankenhuyzen, 2009; Palma et al., 2014). Commercial *B. thuringiensis* powders containing a mixture of toxin crystals and dried spores have already been authorized to be used in organic agriculture and also play an important role in integrated pest management (Lacey et al., 2015). The PPP called XenTari^®^, based on spores of *B. thuringiensis* subspecies *aizawai* ABTS-1857 (BTa ABTS-1857), is one of the most widely used commercial BCAs in the EU for control of caterpillars on fruits, vegetables, nuts, and turf. In the EU the BCAs are placed on the market under the conditions of Regulation (EC) No 1107/2009 although specific registrations for application may vary among countries in the EU.

The plant phyllosphere contains several resident species of microorganisms, and bacteria are typically the most abundant colonizers (Morris and Kinkel, 2002). When enteric human pathogens contaminate plant surfaces, they must co-exist and compete with the resident microbial communities that have already become adapted to the phyllosphere conditions (Brandl, 2006). It is also reported that plant pathogens can assist in the fitness of enteric human pathogens in the phyllosphere by weakening the plant or changing the environment to enhance the survival of microorganisms. These environmental changes include leakage from tissues to increase nutrients and water availability in the plant (Aruscavage et al., 2006). The application of commercial *B. thuringiensis* products on leafy greens also provides an opportunity to colonize the phyllosphere, turning *B. thuringiensis* a part of the local microbial communities.

Some studies have evaluated the occurrence of *B. thuringiensis* and its residues on fresh vegetables either before or after harvest (Pedersen et al., 1995; Haddad et al., 2005; Frederiksen et al., 2006; Stephan et al., 2014; Kim et al., 2017). However, in most of these publications, reports of recovery of *B. thuringiensis* followed the methodology used in chemical deterioration studies, expressing the decrease as a percentage of absolute numbers instead of referring to log reductions as is usual in food microbiology. Yet, none of these studies evaluated the possible interactions between *B. thuringiensis* strains and enteric pathogens associated with foodborne outbreaks on agricultural crops and post-harvest.

*Bacillus thuringiensis* biopesticides are usually administered as dry powders, containing *B. thuringiensis* in the sporulated form. Thus, the effect of commercial *B. thuringiensis* products on the safety of vegetables destined to human consumption as foods depends on the evaluation of the behavior of these spores in the presence of other microorganisms in these vegetables. With this in mind, this study was designed to assess the growth potential of *B. thuringiensis* versus other *B. cereus* group strains and *S. enterica* strains in culture media at various environmental temperatures (4, 7, 12, and 22°C) and then investigate the growth or survival of BTa ABTS-1857 and *S. enterica* serotype Montevideo in inoculated spinach plants (pre-harvest) and cut leaves (post-harvest), as single or co-cultures, taking into account the natural microbiota present. The commercial PPP XenTari^®^ composed by *B. thuringiensis* spores was selected for the purpose of this study.

## Materials and Methods

### Bacterial Strains

The study was conducted with six strains of *S. enterica* and six strains of the *B. cereus* group from the culture collection of the Laboratory of Food Microbiology and Food Preservation (LFMFP) at Ghent University (UGent; Ghent, Belgium) (Table 1). All strains were stored at −75°C on glass beads and revived in 9 ml of brain heart infusion broth (BHI; Oxoid, United Kingdom) overnight at 37°C (*S. enterica*) and 30°C (*B. cereus*) before use. Loops of the overnight cultures were streaked on the surface of Xylose Lysine Deoxycholate (XLD) agar (Oxoid) or Mannitol Egg Yolk Polymyxin (MYP) agar (Oxoid) plates to check the purity of *Salmonella* and *B. cereus* group strains, respectively. After incubation for 24 h, a single colony on each selective agar plates (XLD or MYP) was transferred to BHI slants [37 g/L BHI and 16 g/L bacteriological agar (Oxoid)] and incubated for 24 h at 30°C or 37°C, and kept as work stocks for maximum 6 weeks.

**TABLE 1:** Strains of *Salmonella enterica* and *Bacillus* spp. used in the study.

Strain	LFMFP number	Collection number	Origin
*Bacillus cereus*	836	ATCC 14579	–
	710	–	Mashed potatoes (diarrheal toxin producing strain)
*Bacillus thuringiensis*	464	ATCC 10792	Mediterranean flour moth
	BTa ABTS-1857	SD-1372	XenTari^® #^
*Bacillus weihenstephanensis*	472	LMG 18989	Pasteurized milk
*Bacillus mycoides*	1053	ATCC 6462	Soil
*Salmonella enterica* Thompson	688	RM1987^§^	Cilantro
*Salmonella enterica* Typhimurium	689	ATCC SL 1344	–
*Salmonella enterica* monophasic Typhimurium	1006	S1006	Pig carcass
*Salmonella enterica* Enteritidis	1023	ATCC BAA 1045	Almond
*Salmonella enterica* Montevideo	1024	ATCC BAA 710	Tomato
*Salmonella enterica* Senftenberg	1025	ATCC 43845	–

*^#^Kindly provided by Valent BioSciences LCC (Libertyville, IL, United States).^§^Kindly donated by Dr. Maria Brandl (USDA-ARS, Albany, CA, United States).*

The PPP XenTari^®^ WG was kindly provided by Valent BioSciences LCC (Libertyville, IL, United States), for experimental testing. The XenTari^®^ WG selected for the study was the Water Dispersible Granule powder formulation that contains spores of BTa ABTS-1857 and insecticidal toxin proteins. For BTa ABTS-1857 spores inoculum, the powder was dissolved in sterile distilled water and further diluted until the desired concentration. For BTa ABTS-1857 vegetative cells inoculum, the *B. thuringiensis* strain was isolated from the XenTari^®^ WG powder by plating the dissolved product on MYP agar and incubating for 24 h at 30°C. One single colony was selected, streaked on MYP agar, and then grown on BHI slants at 30°C for 24 h and kept as work stocks for maximum 6 weeks.

### Growth Assessment in BHI

Initially, the growth potential in BHI at 12°C (refrigeration abuse temperature) and 22°C (room temperature) of the twelve strains was assessed in 96-well plates by optical density (OD) measurements at 600 nm in a VersaMax microplate reader (Molecular Devices, San Jose, CA, United States). Briefly, 200 μL/well BHI broth inoculated with ca. 100 CFU/mL bacteria of each strain was added to 96-well plates, and BHI broth without inoculation was used as the negative control. The 96-well plates were inoculated statically, and the OD values of the negative control were subtracted to accomplish expression of the final result, which corresponded to start OD values as 0 at time point 0. At 12°C, the OD was measured once every 12 h up to 120 h (5 days), and at 22°C, the OD measurements were performed at time 0 and 12 h, and then every hour up to 24 h, and a final measurement at 36 h. In the next step, the growth potential of the strains in BHI at 4 ± 1°C, 7 ± 1°C, and 12 ± 1°C was tested in 12-well cell culture plates (SPL Life Sciences Co., Ltd., South Korea) containing 2 mL of BHI in each well. The growth was monitored by spread-plating on XLD (*S. enterica*) or MYP (*Bacillus*) agar and counting of typical colonies on days 0, 5, 9, and 14. These temperatures were selected to simulate different post-harvest low storage temperatures of vegetables. Results were expressed as log CFU/mL ± SD. The maximum growth rates μ_max_ (h^–1^) were derived from the slope of the growth curves in the exponential phase (Zwietering et al., 1990; Widdel, 2007).

### Interactions Between BTa ABTS-1857 and *S.* Montevideo 1024 in BHI

Possible interactions between BTa ABTS-1857 and *S*. Montevideo 1024 were evaluated in BHI at 12°C and 22°C. Cultures were prepared as described in item 2.1 and transferred to 12-well cell culture plates (SPL Life Sciences Co., Ltd., South Korea) containing 2 mL BHI in each well, as single cultures and as co-cultures (ca. 3 log CFU/mL for each bacterial species). Bacterial growth was monitored by plating on MYP for BTa ABTS-1857 and XLD for *S.* Montevideo 1024. Typical colony counts were performed every day up to 5 days.

### Pre-harvest Growth of BTa ABTS-1857 and *S.* Montevideo 1024 on Spinach Plants

#### Spinach Cultivation

Spinach seeds (Maxeda DIY B.V., Netherlands) were sown in plastic trays containing organic soil (Central Park^®^, Maxeda DIY Group, Netherlands) for germination and kept moist by irrigation every 2 days. After 15 days, germinated seedlings were transferred to pots (40 cm × 13 cm × 16 cm, 10 seedlings per pot) containing approximately 2.2 kg of organic soil. Spinach plants were grown inside an indoor grow chamber (Mammoth Lite 90, Netherlands) and irrigated carefully to avoid splashing water and soil onto leaves. A photoperiod of 12 h was ascertained by a 250 W lamp (Sonlight Agro, United Kingdom) in the growth chamber. Temperature 22 ± 3°C and relative humidity (RH) 65 ± 14% were monitored during the full growth period by an EL-USB-2-LCD + logger (Lascar Electronics Ltd., United Kingdom). The plants were grown under these conditions for approximately 6 weeks.

#### Inoculation of Spinach Plants With BTa ABTS-1857 and *S*. Montevideo 1024

In a biosafety cabinet, 54 samples of spinach plants were sprayed (0.5 mL/plant; Pharma-Pack, Antwerp, Belgium; 18 mm) with *S*. Montevideo 1024 and/or BTa ABTS-1857 (vegetative cells or spores), according to six scenarios (nine plants per scenario): (1) sterile distilled water (control); (2) *S*. Montevideo 1024; (3) vegetative cells of BTa ABTS-1857; (4) spores of BTa ABTS-1857; (5) vegetative cells of BTa ABTS-1857 and *S*. Montevideo 1024; (6) spores of BTa ABTS-1857 and *S*. Montevideo 1024. The inoculum of *S*. Montevideo 1024 was 3–4 log CFU/g, selected based on the infectious dose of *Salmonella* spp. in the range of 10,000 cells (Franz and van Bruggen, 2008) and capability of enumeration in case of die-off. The inoculum of vegetative cells of BTa ABTS-1857 was ∼6 log CFU/g, selected as 5 log CFU/g is a potential risk to human health (EFSA BIOHAZ Panel, 2005, 2016). The inoculum of spores of BTa ABTS-1857 was ∼8 log CFU/g. Before use, XenTari^®^ WG was diluted to reach the final concentrations of *B. thuringiensis* spores of ∼8 log CFU/g and ∼6 log CFU/g in the pre- and post-harvest simulations, respectively, mimicking worst-case high concentration immediately after BCA’s spraying. After spraying, the 54 plants were kept inside the biosafety cabinet for 1 h to ensure microbial adhesion to the leaves (Cálix-Lara et al., 2014) and then transferred to a growth chamber, maintaining the same culture conditions as before spraying.

#### Evaluation of Persistence of Vegetative Cells and Spores of BTa ABTS-1857 and *S*. Montevideo 1024 on Spinach Plants

On days 0, 5, and 10 of cultivation (refer to section “Inoculation of Spinach Plants With BTa ABTS-1857 and *S.* Montevideo 1024”), the leaves of three spinach plants per studied scenario were cut and transferred separately into bags (leaves of one plant into one bag) with filters (Bioreba, Switzerland), weighed and mixed with 10 × Buffered Peptone Water (BPW, Oxoid) using a manual homogenizer (Bioreba, Switzerland). Homogenates (triplicates coming from three different spinach plants) were submitted to decimal dilutions in Peptone Physiological Solution (PPS) [1 g/L peptone (Oxoid, Oxford, United Kingdom) + 8.5 g/L NaCl (Sigma-Aldrich, St. Louis, Mo, United States)] and plated on XLD and MYP agar and incubated at 37°C for 24 h for enumeration of *Salmonella* and at 30°C for 24 h for enumeration of presumptive *B. cereus* (which includes *B. thuringiensis*), respectively. For the enumeration of spores of BTa ABTS-1857, the homogenates were heated at 80°C for 10 min before plating on MYP plates. As more spinach plants were available, the persistence of vegetative cells and spores of BTa ABTS-1857 during plant cultivation was also monitored on days 15 and 20 (three plants per treatment). In addition, three samples of 10 g of organic soil used for plant cultivation were weighed before planting, mixed with 10 × BPW, submitted to decimal dilutions in PPS and plated on MYP for the enumeration of presumptive *B. cereus*. Plates were incubated at 30°C for 24 h and typical colonies were counted. Results were expressed as log CFU/g ± SD.

### Post-harvest Growth of BTa ABTS-1857 and *S. enterica* on Spinach Cut Leaves

Packages of pre-washed spinach cut leaves were purchased in a local supermarket in the city of Ghent, Belgium, transported to the laboratory and stored at 6°C for a maximum of 2 h. After mixing the content of the packages in a sterile recipient, 300 g of the mixed leaves were transferred to sterile stomacher bags and spray-inoculated with 3 mL of the inoculum (10 μL/g leaves) in a biosafety cabinet, following the same inoculation scenarios described in section “Inoculation of Spinach Plants With BTa ABTS-1857 and *S.* Montevideo 1024.” After inoculation, bags were closed and stored at room temperature for 60 min for microbial attachment to the leaves (Cálix-Lara et al., 2014), then placed in an airtight box and stored in a refrigerator at 12°C, selected as a maximum refrigerator abuse temperature where the growth of both *Salmonella* and *B. thuringiensis* is still possible. Three portions of 15 g of leaves from each bag were removed daily from the first to the 5th day of storage, transferred individually to stomacher bags, added 135 mL of BPW and homogenized in the stomacher for 1 min. Samples were submitted to decimal dilution in PPS and spread-plated on XLD and MYP plates and incubated at 37°C and 30°C for 24 h, for enumeration of *Salmonella* and presumptive *B. cereus*, respectively. Experiments were done in triplicate and results were expressed as log CFU/g ± SD.

### Distinction of *B. thuringiensis* From Presumptive *B. cereus* on the Spinach Cut Leaves and Soils by Phase-Contrast Microscopy

The presumptive *B. cereus* colonies in MYP-plates isolated from non-*B. thuringiensis* treated spinach samples (controls and leaves sprayed with *S*. Montevideo alone as described in section “Post-harvest Growth of BTa ABTS-1857 and *S. enterica* on Spinach Cut Leaves”) and from soil samples (as described in section “Evaluation of Persistence of Vegetative Cells and Spores of BTa ABTS-1857 and *S.* Montevideo 1024 on Spinach Plants”) were submitted to phase-contrast microscopy (Leica, Germany) for detection of insecticidal crystals and distinction between *B. cereus sensu stricto* and *B. thuringiensis*, as recommended by the Food and Drug Administration (Tallent et al., 2001) described in FDA-BAM^1^ and the International Organization for Standardization (ISO) (ISO 7932:2004/AMD 1:2020, 2020). For the detection of parasporal crystals, 100 μL of overnight cultures in BHI were transferred to strengthened Nutrient Agar (sNA) plates, containing 28 g/L Nutrient Agar (Oxoid), 0.04 g/L MgCl_2_ (Sigma-Aldrich), and 0.10 g/L CaCl_2_ (Sigma-Aldrich). The colonies in the sNA plates were monitored by phase-contrast microscopy until they reached the sporulation stage (approximately 24–48 h incubation). The sporulating cells were examined before lysis of the mother cells.

### Statistical Analysis

All statistical tests were performed in SPSS Statistics 26 software (IBM SPSS Statistics, NY, United States). The normality and equality of variances were checked by Shapiro–Wilk test or Q–Q plot and Levene’s test, respectively. Thereafter, differences within each treatment among days were analyzed by one-way analysis of variance (ANOVA) (α = 0.05), followed by the Tukey honestly significant difference (HSD) as a *post hoc* test when the equality of variances was confirmed. When equality of variances was not verified, one-way ANOVA followed by the Games-Howell *post hoc* test was used. Statistical significant difference was indicated by a *p* value of less than 0.05. In addition to the analysis to indicate statistical significant difference, the results on increase or decrease of colony counts in time or growth potential were also compared to a threshold value of 0.5 log unit, that indicates the relevance of increase or decrease in microbiological colony count data (Jarvis et al., 2007; Uyttendaele et al., 2018; ISO 19036, 2019).

## Results

### Growth Rate of *B. cereus* Group and *S. enterica* Strains at Cold and Room Temperatures

**FIGURE 1:**
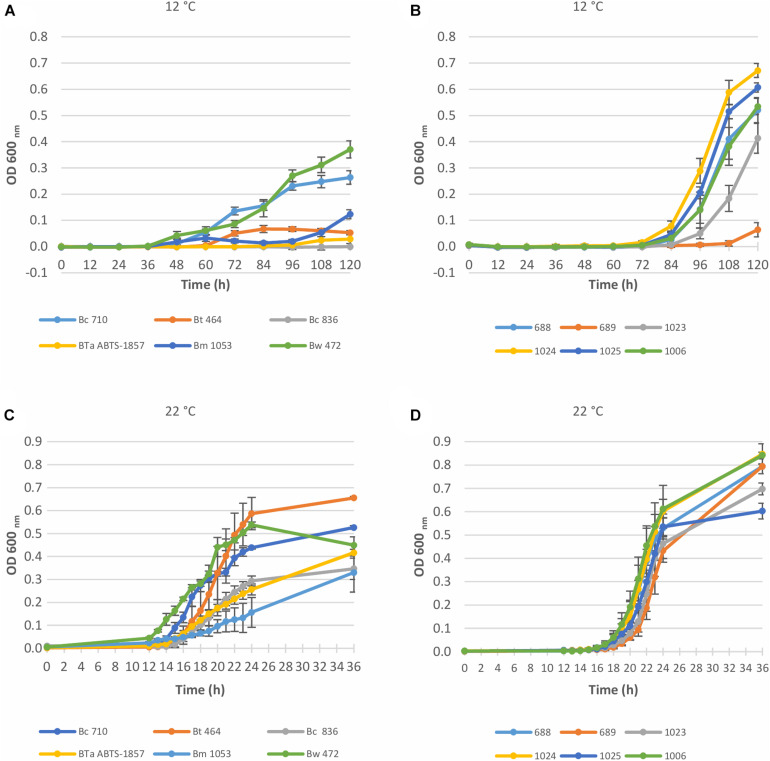
Growth curves of *Bacillus cereus* group and *S. enterica* strains in BHI at 12°C and 22°C, determined by OD measurement at 600 nm. Data represent the mean of six replicates ± SD. **(A)** 6 *Bacillus cereus* group strains at 12°C; **(B)** 6 *S. enterica* strains at 12°C; **(C)** 6 *Bacillus cereus* group strains at 22°C; **(D)** 6 *S. enterica* strains at 22°C. Bw, *B. weihenstephanensis*; Bm, *B. mycoides*; BTa ABTS-1857, *B. thuringiensis* subsp. *aizawai* ABTS-1857; Bt, *B. thuringiensis*; Bc, *B. cereus*; 688, *S. enterica* Thompson; 689, *S. enterica* Typhimurium; 1006, *S. enterica* monophasic Typhimurium; 1023, *S. enterica* Enteritidis; 1024, *S. enterica* Montevideo; 1025, *S. enterica* Senftenberg.

Growth of *B. cereus* group and *S. enterica* strains in BHI at 12°C (cold) and 22°C (ambient) is shown in Figure 1. As expected, all tested strains grew better at 22°C than at 12°C. Because of the limitation of OD measurements, in which readings may be affected by the type and morphology of the bacterial cells, by their tendency to form clusters or aggregates, and by their capability to form biofilm in the bottom of the microplates, the growth capability was also measured by plating in appropriate culture media at 12°C (Tables 2, 3). No change could be detected in the OD measurements for Bc 836 in 5 days at 12°C. Enumeration results of Bc 836 at 12°C on day 5 were below 6 log CFU/mL (Table 2), which is below the threshold level of the VersaMax microplate reader at 600 nm, which could explain the lack of change in the baseline by OD measurement. The two types of growth measurements proved to be complementary due to the small size of the initial inoculum. 12°C is generally considered in the EU as the maximum foreseen temperature of temperature abuse at the consumer’s home (EURL Lm, 2014), deviating from the standard refrigerator’s temperature in retail or consumer stage ranging from 4 to 8°C (Xanthiakos et al., 2006; Roccato et al., 2017). Therefore, the growth potential was also assessed at two more cold temperatures (4°C and 7°C) monitoring the growth potential by plating (Tables 2, 3). The μ_max_ (h^–1^) values of all tested strains at different temperatures are shown in Table 4. The μ_max_ values were higher at 22°C than in the other tested temperatures, both for *B. cereus* group and *S. enterica* strains.

**TABLE 2:** Counts of *B. cereus* group strains in BHI at 4°C, 7°C, and 12°C, determined by plate count on MYP agar.

Temperature	Day	Bw 472	Bm 1053	BTa ABTS-1857	Bt 464	Bc 836	Bc 710
4°C	0	2.15 ± 0.02	1.76 ± 0.14	1.85 ± 0.31	1.71 ± 0.12	1.61 ± 0.04	1.51 ± 0.06
	5	3.33 ± 0.24	2.68 ± 0.17	3.15 ± 0.26	2.10 ± 0.17	2.65 ± 0.43*	1.40 ± 0.29
	9	1.95 ± 0.07	2.90 ± 0.05	1.59 ± 0.05	0.80 ± 0.17	1.46 ± 0.21	0.56 ± 0.24
	14	0.69 ± 0.09	3.15 ± 0.48	0.45 ± 0.21*	1.00 ± 0.00*	1.22 ± 0.17	NA
7°C	0	2.15 ± 0.02	1.76 ± 0.14	1.85 ± 0.31	1.71 ± 0.12	1.61 ± 0.04	1.51 ± 0.06
	5	6.40 ± 0.29	5.42 ± 0.24	1.00 ± 0.00	2.81 ± 0.07*	1.10 ± 0.17	3.58 ± 0.21
	9	7.00 ± 0.02	7.07 ± 0.10	0.95 ± 0.33	1.00 ± 0.00**	0.82 ± 0.20	4.59 ± 0.11
	14	6.79 ± 0.07	6.68 ± 0.08	0.96 ± 0.23	0.90 ± 0.00**	0.30 ± 0.00	5.18 ± 0.11
12°C	0	2.15 ± 0.02	1.76 ± 0.14	1.85 ± 0.31	1.71 ± 0.12	1.61 ± 0.04	1.51 ± 0.06
	5	9.15 ± 0.00	6.22 ± 0.14	6.20 ± 0.35	8.49 ± 0.00	3.54 ± 0.34	7.76 ± 0.13
	9	8.46 ± 0.24	7.20 ± 0.45	5.53 ± 0.14	7.71 ± 0.07	3.51 ± 0.26	8.04 ± 0.08
	14	8.82 ± 0.04	7.53 ± 0.09	5.92 ± 0.18	8.31 ± 0.03	3.26 ± 0.63	8.11 ± 0.10

Data represent the mean of triplicates ± SD (log CFU/mL).* and ** indicate, respectively, one and two out of three samples are below the detection limit.NA means no available data, all triplicates are below the detection limit.Bw, B. weihenstephanensis; Bm, B. mycoides; BTa ABTS-1857, B. thuringiensis subsp. aizawai strain ABTS-1857; Bt, B. thuringiensis; Bc, B. cereus.

**TABLE 3:** Counts of *S. enterica* strains in BHI at 4°C, 7°C, and 12°C, determined by plate count on XLD agar.

Temperature	Day	*S.* Thompson 688	*S.* Typhimurium 689	*S.* monophasic Typhimurium 1006	*S.* Enteritidis 1023	*S.* Montevideo 1024	*S.* Senftenberg 1025
4°C	0	1.24 ± 0.02	1.27 ± 0.05	1.31 ± 0.05	1.30 ± 0.09	1.30 ± 0.06	1.23 ± 0.07
	5	1.50 ± 0.13	1.78 ± 0.01	1.54 ± 0.19	1.62 ± 0.07	1.52 ± 0.05	1.31 ± 0.17
	9	0.89 ± 0.14	1.77 ± 0.07	1.52 ± 0.12	1.72 ± 0.18	1.62 ± 0.06	1.45 ± 0.08
	14	0.71 ± 0.41	1.39 ± 0.08	1.37 ± 0.05	1.56 ± 0.12	1.22 ± 0.24	1.12 ± 0.10
7°C	0	1.24 ± 0.02	1.27 ± 0.05	1.31 ± 0.05	1.30 ± 0.09	1.30 ± 0.06	1.23 ± 0.07
	5	1.95 ± 0.13	2.03 ± 0.15	1.22 ± 0.07	2.06 ± 0.04	2.05 ± 0.04	1.99 ± 0.08
	9	1.89 ± 0.09	2.02 ± 0.02	0.98 ± 0.05	2.07 ± 0.23	2.20 ± 0.07	1.91 ± 0.04
	14	0.85 ± 0.71	1.32 ± 0.14	0.26 ± 0.24	1.95 ± 0.15	1.70 ± 0.10	1.10 ± 0.28
12°C	0	1.24 ± 0.02	1.27 ± 0.05	1.31 ± 0.05	1.30 ± 0.09	1.30 ± 0.06	1.23 ± 0.07
	5	9.03 ± 0.13	8.71 ± 0.02	8.99 ± 0.02	9.12 ± 0.08	9.19 ± 0.02	8.98 ± 0.17

*Data represent the mean of triplicates ± SD (log CFU/mL).*

**TABLE 4:** Maximum growth rate (μ_max_, h^–1^) of *B. cereus* group strains and *S. enterica* strains at different temperatures.

Bacterium	4°C	7°C	12°C		22°C
	μ_max (VC)_	μ_max (VC)_	μ_max (VC)_	μ_max (OD)_	μ_max (OD)_
*B. weihenstephanensis* 472	0.023	0.082	0.134	0.253	0.542
*B. mycoides* 1053	0.018	0.070	0.086	0.302	0.414
BTa ABTS-1857	0.025	−0.016	0.083	0.114	0.803
*B. thuringiensis* 464	0.007	0.021	0.130	0.210	0.868
*B. cereus* 836	0.020	−0.010	0.037	NA	0.910
*B. cereus* 710	−0.002	0.040	0.120	0.130	0.755
*S*. Thompson 688	0.006	0.015	0.150	0.300	0.738
*S*. Typhimurium 689	0.010	0.013	0.142	0.139	0.689
*S*. monophasic Typhimurium 1006	0.004	−0.002	0.148	0.158	0.648
*S*. Enteritidis 1023	0.006	0.015	0.150	0.132	0.692
*S.* Montevideo 1024	0.004	0.015	0.152	0.182	0.702
*S.* Senftenberg 1025	0.002	0.015	0.150	0.185	0.834

*μ_max (VC)_, calculate based on viable count measurements; μ_max (OD)_, calculate based on OD measurements.NA, no available data due to no changes of OD values of Bc 836 were detected.*

For the *B. cereus* group, the tested strains varied in the capability to grow in BHI at 12°C. As shown in Figure 1A and Table 2, the type strain of *B. weihenstephanensis* (Bw 472) and the foodborne diarrheal toxin producing *B. cereus* strain (Bc 710), isolated from refrigerated mashed potatoes, were the two strains with the best growth potential at 12°C. It is also worth noting that the growth of BTa ABTS-1857 at 12°C was less evident than *B. thuringiensis* type strain Bt 464 (Table 2).

As occurred at 12°C, OD values measured at 22°C indicated variability in the growth potential among the *B. cereus* group strains. Figure 1C indicates that the growth rate at 22°C of *B. thuringiensis* type strain Bt 464 and Bw 472 and Bc 710 was high, whereas BTa ABTS-1857 showed slower growth, similar to the *B. cereus* type strain Bc 836.

The type strains *B. weihenstephanensis* (Bw 472) and *B. mycoides* (Bm 1053) were able to grow in BHI at 7°C, and Bm 1053 presented growth even at 4°C (Table 2). Among the *B. cereus* strains only Bc 710, which is a diarrheal toxin producing strain, was capable to grow at 7°C. The counts of both *B. thuringiensis* strains in BHI after 14 days at 7°C were 0.5 log lower than the initial inoculum, indicating that these two strains are not psychrotrophic or cold tolerant.

For the tested *S. enterica* strains, no substantial outgrowth (>0.5 log growth potential) was observed at 4°C or 7°C after 14 days (Table 3), except for *S*. Enteritidis with a slight growth (ca. 0.6 log) was noted at 7°C after 14 days. However, at 12°C a fast outgrowth of all *S. enterica* strains was noted, reaching counts above 8 log CFU/mL in 5 days. At this temperature, no more readings were necessary. As *S*. Montevideo 1024 presented the best growth potential at both 12°C and 22°C in BHI (Figures 1B,D and Table 3) this strain was selected for the next experiments.

Results of the interactions between BTa ABTS-1857 and *S*. Montevideo 1024 investigated in BHI before the pre and post-harvest simulation are shown in Table 5. At 22°C, no interaction was observed within 5 days, as counts of single cultures were similar to counts in co-cultures. However, at 12°C the counts of BTa ABTS-1857 on the 5th day were 1.5 log lower when in co-culture with *S*. Montevideo 1024 than when in single culture (*p* < 0.05). Despite the statistical significance of the difference in the enumeration results of *S*. Montevideo 1024 grown in co-culture with BTa ABTS-1857 compared to single pure culture at 12°C, the observed differences (<0.5 log reduction) have little, if any, biological relevance. These results indicate that *Salmonella* suppressed the growth of the biopesticide *B. thuringiensis* strain to some extent in BHI at 12°C but not at 22°C.

**TABLE 5:** Counts of BTa ABTS-1857 and *S.* Montevideo 1024 in BHI incubated at 12°C and 22°C for 5 days, as single and co-cultures.

Temperature	Day	BTa ABTS-1857 (single culture)	BTa ABTS-1857 (co-culture with *S.* Montevideo 1024)	*S.* Montevideo 1024 (single culture)	*S.* Montevideo 1024 (co-culture with BTa ABTS-1857)
12°C	0	3.01 ± 0.06	3.01 ± 0.06	3.91 ± 0.05	3.91 ± 0.05
	1	3.45 ± 0.11^A^	3.40 ± 0.11^A^	5.90 ± 0.07^a^	5.47 ± 0.18^a^
	5	5.74 ± 0.06^A^	4.12 ± 0.11^B^	9.39 ± 0.06^a^	9.18 ± 0.05^b^
22°C	0	3.01 ± 0.06	3.01 ± 0.06	3.91 ± 0.05	3.91 ± 0.05
	1	7.60 ± 0,18^A^	7.17 ± 0.07^A^	9.22 ± 0.08^a^	9.09 ± 0.12^a^
	5	7.74 ± 0.65^A^	7.56 ± 0.07^A^	10.06 ± 0.13^a^	9.82 ± 0.03^a^

*Mean of triplicate samples ± SD (log CFU/mL). Different superscript letters denote significant difference (p < 0.05) when comparing BTa ABTS-1857 or S. Montevideo 1024 in single culture and co-culture on the same day at 12°C or 22°C.*

### Pre-harvest Growth of BTa ABTS-1857 and *S.* Montevideo 1024 on Spinach Plants

**FIGURE 2:**
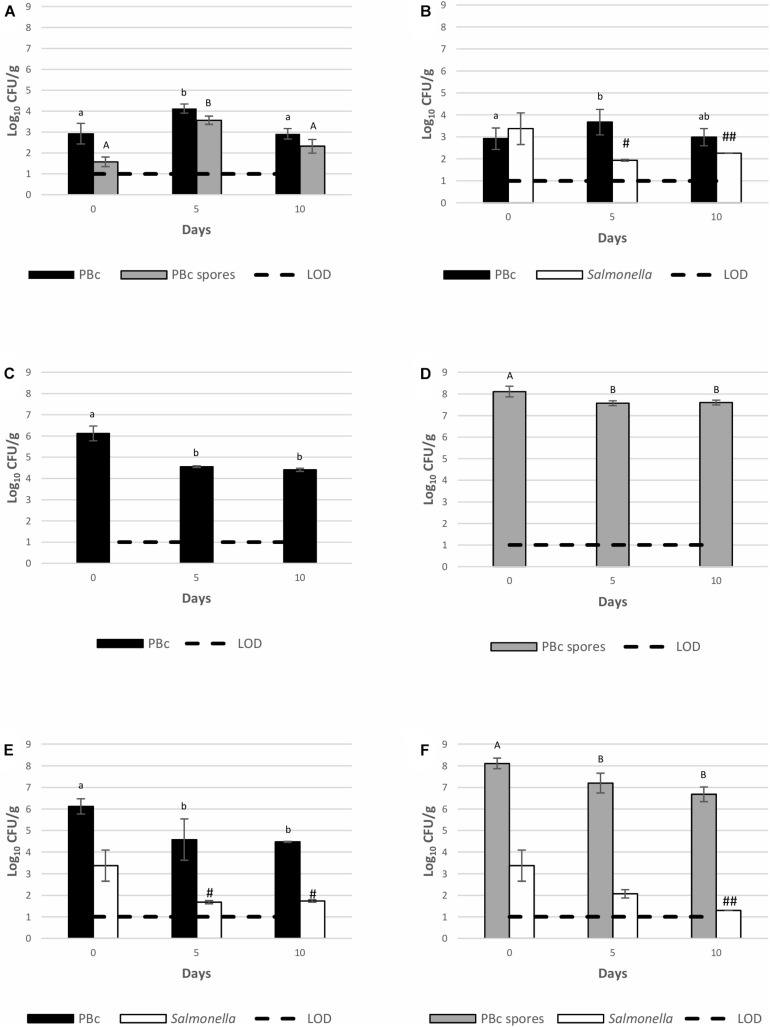
Behavior of *S*. Montevideo 1024 and BTa ABTS-1857 as single and co-cultures on spinach plants during cultivation. **(A)** controls (plants sprayed with sterile distilled water); **(B)** plants sprayed with *S*. Montevideo 1024; **(C)** plants sprayed with vegetative cells of BTa ABTS-1857; **(D)** plants sprayed with spores of BTa ABTS-1857; **(E)** plants sprayed with vegetative cells of BTa ABTS-1857 and *S*. Montevideo 1024; **(F)** plants sprayed with spores of BTa ABTS-1857 and *S*. Montevideo 1024. The bars represent the mean of triplicates ± SD. Horizontal dashed lines indicate the detection limits. PBc, presumptive *B. cereus* (counts on MYP prior to heat treatment, i.e., both vegetative cells and spores); PBc spores, presumptive *B. cereus* spores (counts on MYP after heat treatment). # and ## indicate, respectively, one and two out of three samples are <1.0 log_10_ CFU/g. Bars in the same color sharing common letters are not significantly different from each other (*p* ≥ 0.05). Statistical analysis was not possible for *Salmonella* due to several negative results.

Growth of *S*. Montevideo 1024 and BTa ABTS-1857 on spinach plants (22 ± 3°C, 65 ± 14% RH, 12 h daylight) inoculated as single cultures or co-cultures is shown in Figure 2. Temperature and humidity monitored during pre-harvest simulation are shown in Supplementary Table 1. *Salmonella* was absent in control samples where neither *S*. Montevideo 1024 nor BTa ABTS-1857 were sprayed (Figure 2A). However, the counts of presumptive *B. cereus* as natural background varied from 2.9 to 4.1 log CFU/g. When inoculated as single culture (ca. 1000 CFU/g), *S*. Montevideo 1024 populations presented a 1.1 log reduction on the 10th day. However, when co-inoculated with vegetative cells (Figure 2E) and spores (Figure 2F) of BTa ABTS-1857, reduction of 1.6 and 2.1 log on the counts of *S*. Montevideo 1024 were observed in the 10th day, respectively.

The behavior of BTa ABTS-1857 vegetative cells on spinach plants when inoculated as single culture or co-inoculated with *S*. Montevideo 1024 was similar: a mean reduction of 1.6–1.7 log in the counts of presumptive *B. cereus* (PBc, which includes BTa ABTS-1857) was noted on the 10th day (Figure 2E). The counts of spores when added alone presented ca. 0.5 log reduction on the 5th day, but remained stable until the 10th day. The initial 0.5 log reduction in spore counts was hypothesized to be attributed to spinach plants growing larger leaves in the first 5 days with significant differences of cut spinach leaves’ weights shown between day 0 and day 5 (*p* < 0.05, Supplementary Figure 1).

**FIGURE 3:**
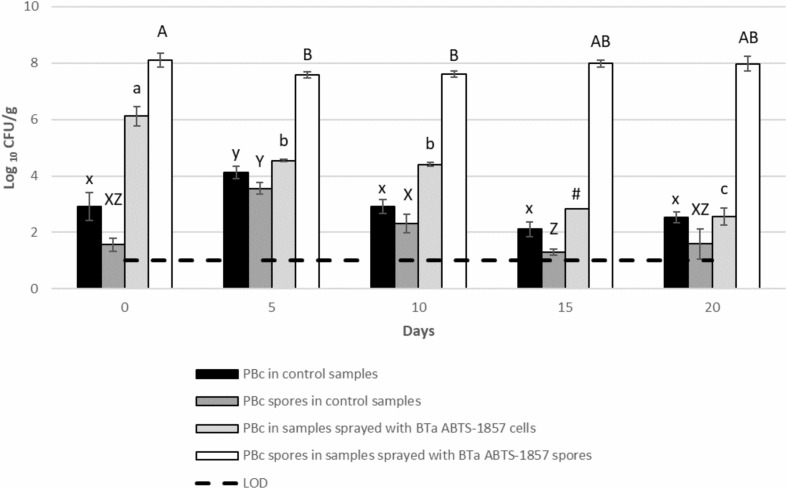
Behavior of vegetative cells and spores of BTa ABTS-1857 on spinach plants during cultivation. Results on day 0, 5, and 10 are the same in Figure 2, but more count results on day 15 and day 20 were shown here. Each data bar represents the mean of triplicate samples (3 spinach plants), and each error bar indicates ± the standard deviation. Horizontal dashed lines indicate the detection limit. Statistical differences of comparisons were analyzed within the same treatment among days. PBc, presumptive *B. cereus* (counts on MYP prior to heat treatment, i.e., both vegetative cells and spores); PBc spores, presumptive *B. cereus* spores (counts on MYP after heat treatment). Bars in the same color sharing common letters are not significantly different from each other (*p* ≥ 0.05). # indicates one out of three samples is <1.0 log_10_ CFU/g.

As shown in Figure 3, a 1.7 log reduction in the counts of BTa ABTS-1857 vegetative cells was observed on the 10th day. Thereafter a further 1.9 log reduction occurred on the 20th day, reaching the benchmark level of the non-inoculated control plants showing the presence of “naturally occurring” presumptive *B. cereus* in the range of 100–1000 CFU/g. On the other hand, the counts of BTa ABTS-1857 spores remained more or less stable, varying between 7.6 and 8.1 log CFU/g within the 20 days. Interestingly, the counts of presumptive *B. cereus* in non-inoculated control spinach plants varied from a maximum of 4.1 log CFU/g on the 5th day to a minimum of 2.1 log CFU/g on the 15th day. For the spore counts of presumptive *B. cereus* in the control samples, a larger variability was observed: the counts varied from 1.3 log CFU/g on the 15th day to 3.6 log CFU/g on the 5th day. It is worth noting that the mean count of presumptive *B. cereus* in the three organic potting soil samples was 3.9 ± 0.1 log CFU/g. In addition, phase-contrast microscopy indicated that 2 out of 12 presumptive *B. cereus* isolates taken from the MYP-plates from the potting soil counts were identified as *B. thuringiensis* strains.

Overall, the results on the behavior in the pre-harvest stage indicated a reduction in the counts of BTa ABTS-1857 vegetative cells and *S*. Montevideo 1024 on spinach leaves. However, persistence of BTa ABTS-1857 spores was noted.

### Post-harvest Growth of BTa ABTS-1857 and *S.* Montevideo 1024 on Spinach Cut Leaves

**FIGURE 4:**
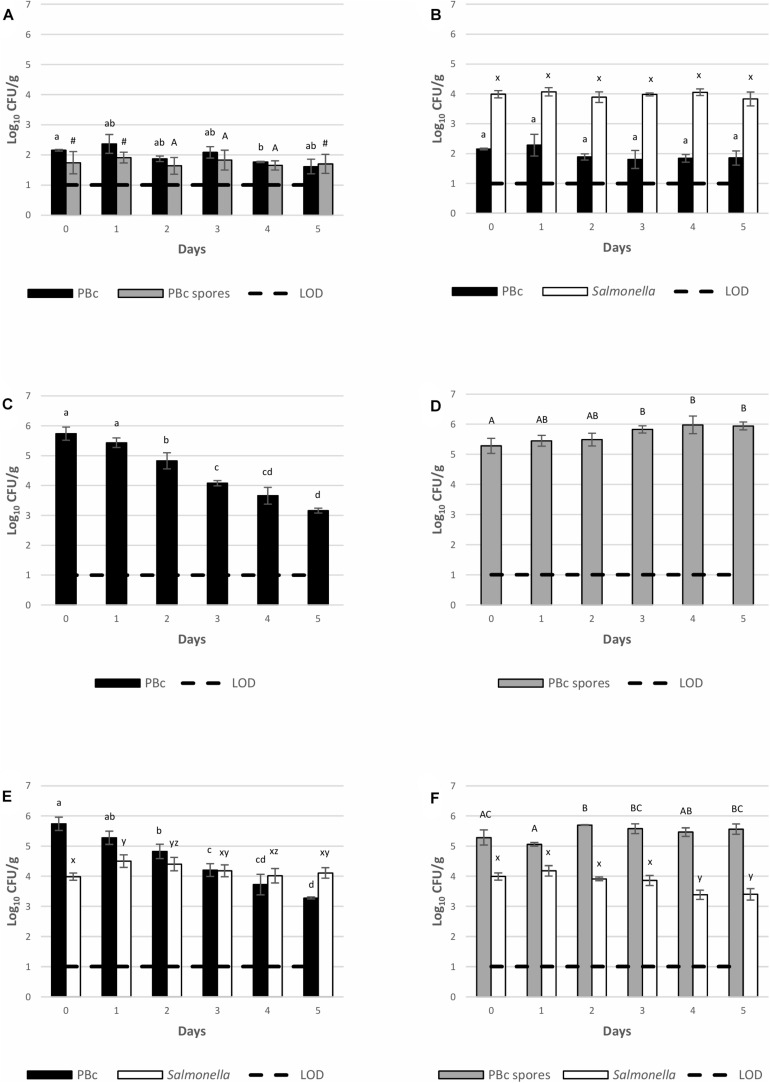
Persistence of *S*. Montevideo 1024 and BTa ABTS-1857 on spinach cut stored at 12°C when inoculated as single and co-cultures. **(A)** controls (cut leaves sprayed with sterile distilled water); **(B)** cut leaves sprayed with *S*. Montevideo 1024; **(C)** cut leaves sprayed with vegetative cells of BTa ABTS-1857; **(D)** cut leaves sprayed with spores of BTa ABTS-1857; **(E)** cut leaves sprayed with vegetative cells of BTa ABTS-1857 and *S*. Montevideo 1024; **(F)** cut leaves sprayed with spores of BTa ABTS-1857 and *S*. Montevideo 1024. Each bar represents the mean of triplicate samples ± SD. Horizontal dashed lines indicate the detection limits. PBc, presumptive *B. cereus* (counts on MYP prior to heat treatment, i.e., both vegetative cells and spores); PBc spores, presumptive *B. cereus* spores (counts on MYP after heat treatment). Bars in the same color sharing common letters are not significantly different from each other (*p* ≥ 0.05). # indicates one out of three samples is <1.0 log_10_ CFU/g.

The capability of BTa ABTS-1857 and *S*. Montevideo 1024 to grow on inoculated spinach cut leaves during 5 days of storage at 12°C is indicated in Figure 4. The count of presumptive *B. cereus* in the bagged spinach cut leaves bought in the supermarket before experimental inoculation (Figure 4A) was 2.2 log CFU/g, and a 0.6 log decrease was observed on the 5th day. The counts of spores (1.8 log CFU/g) from leaves remained almost the same along the 5 days of the experiment. The counts of *S*. Montevideo 1024 on the spinach cut leaves, when inoculated alone (Figure 4B) or co-inoculated with vegetative cells of BTa ABTS-1857 (Figure 4E) or spores of BTa ABTS-1857 (Figure 4F) remained similar to the initial inoculum along the 5 days of storage at 12°C, indicating survival but not growth or death. Only a small reduction of counts of *S*. Montevideo 1024 was observed after 4 days on the leaves inoculated with the spores of BTa ABTS-1857 (Figure 4F). In contrast, more than 2 log reductions occurred in the counts of presumptive *B. cereus* in the leaves inoculated with BTa ABTS-1857 vegetative cells (alone or co-inoculated with *S*. Montevideo 1024) (Figures 4C,E), after 5 days at 12°C. When 40 presumptive *B. cereus* isolates obtained from leaves not treated with BTa ABTS-1857 were subjected to phase-contrast microscopy to differentiate *B. cereus* from *B. thuringiensis*, 28 (70%) were identified as *B. thuringiensis*. Overall, the results on the behavior of BTa ABTS-1857 and *S*. Montevideo 1024 in the post-harvest stage indicated only a reduction of BTa ABTS-1857 vegetative cells, and only on spinach cut leaves stored at 12°C.

## Discussion

*Bacillus cereus* and *B. thuringiensis* have been considered microbiological hazards and further characterized as potential foodborne human pathogens (Gaviria Rivera et al., 2000; Ghelardi et al., 2007; EFSA BIOHAZ Panel, 2016; Johler et al., 2018; BfR, 2019). However, the risk of foodborne poisoning due to the consumption of fresh produce containing *B. thuringiensis* depends on actual exposure at the time of consumption, which depends on the numbers (dose) in the product. Therefore, risk calculations must be based on the entire production chain (farm to fork), taking into account the behavior of *B. thuringiensis* in the pre-harvest (during produce growth), and post-harvest (during cold storage) steps.

The behavior of BTa ABTS-1857 and *S*. Montevideo 1024 in the spinach leaves in the pre-harvest and post-harvest lab simulations differed from what was observed in BHI at 22°C and 12°C. Despite the favorable intrinsic factors in the spinach plant leaves (nutrients availability and neutral pH) and good extrinsic conditions (22°C and 65% RH) for bacterial growth, die-off of *Salmonella* was noted instead of outgrowth. This behavior has also been reported for other plants in pre-harvest phase, such as basil, lettuce, spinach, and tomato leaves (Gu et al., 2011; Li and Uyttendaele, 2018; López-Gálvez et al., 2018; Roy and Melotto, 2019). Additionally, studies with cilantro, romaine and spinach cultivated under similar conditions as in this study (ca. 22°C and 65% RH) indicated a smaller population size of *Salmonella* viable cells with weak fitness in the phyllosphere when compared to cultivation at higher temperature and humidity (e.g., 30°C or 37°C, 95% RH), which are more favorable conditions for growth of enteric bacteria (Brandl and Mandrell, 2002; Brandl, 2006; Roy and Melotto, 2019). The growth of *Salmonella* on plant leaves can be restricted by the transient stomatal closure (Roy and Melotto, 2019; Johnson et al., 2020) and activation of the plant immune response (Meng et al., 2013). Hsu and Micallef (2017) also reported that beneficial *Pseudomonas* strains exhibiting plant growth-promoting rhizobacteria (PGPR) properties reduce the fitness of epiphytic *S. enterica* in the phyllosphere on tomato and spinach leaves. Enterobacteriaceae in counts varying between 3.3 and 5.0 log in some control spinach plant samples in this study may also have restricted the attachment and colonization of *S. enterica* to the leaves. Previous studies have shown that some Enterobacteriaceae (*Enterobacter asburiae*) and *Pseudomonas* species such as *Pseudomonas chlororaphis* possess antagonistic activity toward fungi and may have affected the attachment and colonization of *S. enterica* in the spinach leaves (Thomashow and Weller, 1995; Cooley et al., 2003).

Interestingly, the behavior (die-off) of *Salmonella* inoculated as single culture to the spinach plant leaves in pre-harvest simulation differed from what occurred when *S*. Montevideo 1024 was inoculated onto spinach cut leaves stored at 12°C (post-harvest), where the level of contamination remained stable (10^4^ CFU/g). A hypothesis to explain this is that cut leaves under post-harvest storage are not intact plants, thus more nutrients are available due to leakage/damage from the leaves, supporting a better survival of *Salmonella* when compared to growing plant leaves (pre-harvest). Good survival of *Salmonella* on leafy greens during post-harvest storage at cold temperature was also reported by other researchers (Kroupitski et al., 2013; Waitt et al., 2014; Delbeke et al., 2015). A previous study has reported that nutrients and juices released from the cut ends of the leaves enabled *Salmonella* to grow and helped the attachment of the cells to the leaves, even under refrigeration (Koukkidis et al., 2017). Although 12°C is not restrictive for the multiplication of *Salmonella* cells, the presence of competing epiphytic bacteria on the spinach leaves in high levels, e.g., Enterobacteriaceae (3.1–6.4 log CFU/g) in the spinach cut leaves bought in the supermarkets (data not shown) may have limited the outgrowth of *Salmonella*.

The behavior of BTa ABTS-1857 vegetative cells on spinach leaves, either as single or co-cultures, in pre- and post-harvest conditions, was similar. This result suggests that it is unlikely that vegetative cells will persist during plant growth and further storage under abuse storage temperature of 12°C. However, XenTari^®^ is a powder containing a mixture of Cry-toxin crystals and dried spores, and these spores have shown to be quite stable on the spinach leaves when inoculated in high numbers, either in the pre-harvest or post-harvest simulations. These results differed from what was reported by Stephan et al. (2014), in a study with greenhouse tomatoes sprayed with XenTari^®^. These authors observed that within 1 week the concentration of *B. thuringiensis* spores on the tomatoes was reduced to between 46 and 77% of the initial numbers, but the count of residual *B. thuringiensis* spores still remained high, exceeding 10^4^ CFU/g fresh weight, and less than 1.0-log reduction was observed. This contrasting result may be attributed to differences in the plants physiology and growth conditions: spinach plants were grown under controlled indoor lab simulation whereas tomatoes were grown in a greenhouse environment, which is known to be more prone to microbial contamination from air and irrigation water (Holvoet et al., 2015). Moreover, the weather conditions on the field impacts the composition of the microbial community (Truchado et al., 2017) and survival of enteric pathogens (Allende et al., 2017), and most probably, *B. cereus* group spores as well. It has been shown that solar or UV radiation, temperature, pH, humidity, wind, rain, and foliage physiology limit the persistence of *B. thuringiensis* spores on vegetables (Glare and O’Callaghan, 2000; Brar et al., 2014). Thus, the present indoor pre-harvest lab simulation is in need of further follow-up research being executed in either greenhouse conditions or agricultural field studies.

It is worth to note that presumptive *B. cereus* was naturally present on spinach leaves in both pre-harvest and post-harvest experiments within the range of 2–4 log CFU/g. The finding of presumptive *B. cereus* (some identified as *B. thuringiensis* by phase-contrast microscopy) in the potting soil suggests that the spinach leaves became contaminated by contact with (or spread of) dust in the chamber, coming from this soil. From 40 *B. cereus*-like isolates taken from the spinach cut leaves used in the post-harvest simulations, 28 (70%) were phenotypically confirmed as *B. thuringiensis* strains by phase-contrast microscopy and presence of parasporal crystals. It should be acknowledged that there was no information on whether these spinach leaves (bought in the supermarket) had been treated with *B. thuringiensis* as BCA in the pre-harvest stage, even considering that the use of *B. thuringiensis* as a BCA in these spinach leaves from non-organic production is rare in Belgium. Thus, the agar plate method used for enumeration did not allow the distinction between the XenTari^®^ *B. thuringiensis* and the autochthonous *B. thuringiensis* strains potentially present on the spinach leaves, as noted for the control samples. Only 2 (16.7%) out of 12 isolates of presumptive *B. cereus* collected from the MYP plates of the potting soil (organic soil) used for spinach plant cultivation in the pre-harvest simulation were identified as *B. thuringiensis* strains by phase-contrast microscopy. Further tests using molecular techniques are needed to identify these isolates and determine if they are naturally occurring soil-dwelling or biocontrol *B. thuringiensis* strains.

In conclusion, the results of the present study indicate that *B. thuringiensis* strains, including BTa ABTS-1857, are not able to grow at the recommended maximum refrigeration temperature of 7°C. Despite variable rate of growth of the *Salmonella* strains and apparently better growth than *B. thuringiensis* strains in BHI at 12°C, there is no reason for concern for spinach leaves: neither *B. thuringiensis* vegetative cells nor *Salmonella* cells grew on the leaves in both pre-harvest and post-harvest simulation studies. Instead, a die-off of *B. thuringiensis* vegetative cells and *Salmonella* was observed, with a possible survival of *Salmonella* during post-harvest storage and persistence of *B. thuringiensis* biopesticide spores during both pre- and post-harvest storage. In the pre-harvest simulation, *S*. Montevideo counts decreased more rapidly when co-inoculated with BTa ABTS-1857 vegetative cells or spores, but this phenomenon was not observed on cut spinach leaves during post-harvest storage.

The persistence of high numbers of *B. thuringiensis* spores in leafy greens in both pre- and post-harvest stages might be similar to that of *B. cereus* sensu stricto, a food poisoning agent and a possible threat for public health (EFSA BIOHAZ Panel, 2016; Raymond and Federici, 2017). Thus, this study highlights the importance of appropriate use of this BCA in crops to prevent residual high numbers of *B. thuringiensis* being present on the leafy greens upon consumption. *B. thuringiensis* can be present in these products in consequence of the use of commercial XenTari^®^, but *B. thuringiensis* can also be present as natural contaminant as a common soil-dwelling bacterium in the agricultural environment. This study helps to understand how naturally occurring *B. thuringiensis* and *B. thuringiensis* in XenTari^®^ behave in the farm to fork chain, providing information for better calculating the human exposure at the time of consumption. Furthermore, the better survival of *S*. Montevideo 1024 during the post-harvest phase compared to the pre-harvest phase is of higher concern. However, there are limitation to this study as only one strain per species was used in the experiments. More data are necessary for the construction of a comprehensive and differentiated risk assessment with regard to microbiological safety of fresh produce. Such data can be applied in the development of microbiological risk assessment models, thus generating estimates of the impact of agricultural practices and post-harvest storage of leafy greens on the microbiological safety of the vegetable production in organic production or integrated pest management when avoiding the use of synthetic pesticides and promoting biological control agents.

## Data Availability Statement

The original contributions presented in the study are included in the article/Supplementary Material, further inquiries can be directed to the corresponding author.

## Author Contributions

XZ and MS performed the experiments and data analysis and prepared the manuscript. IV helped in the experimental design and in cultivation of the spinach plants. MU and BF conceived and designed the study and revised the manuscript. All authors contributed to the article and approved the submitted version.

## Conflict of Interest

The authors declare that the research was conducted in the absence of any commercial or financial relationships that could be construed as a potential conflict of interest.

## Footnotes

1 https://www.fda.gov/food/laboratory-methods-food/bam-bacillus-cereus
