# Supplementary material for: Behavior of the Biological Control Agent Bacillus thuringiensis subsp. aizawai ABTS-1857 and Salmonella enterica on Spinach Plants and Cut Leaves
Source: Front Microbiol. 2021 Feb 3;12:626029. doi: 10.3389/fmicb.2021.626029 (PMC7886684; doi:10.3389/fmicb.2021.626029)
Supplement: Supplementary file 1 [file Image_1.PDF]

Supplementary Material

1 Supplementary Figures

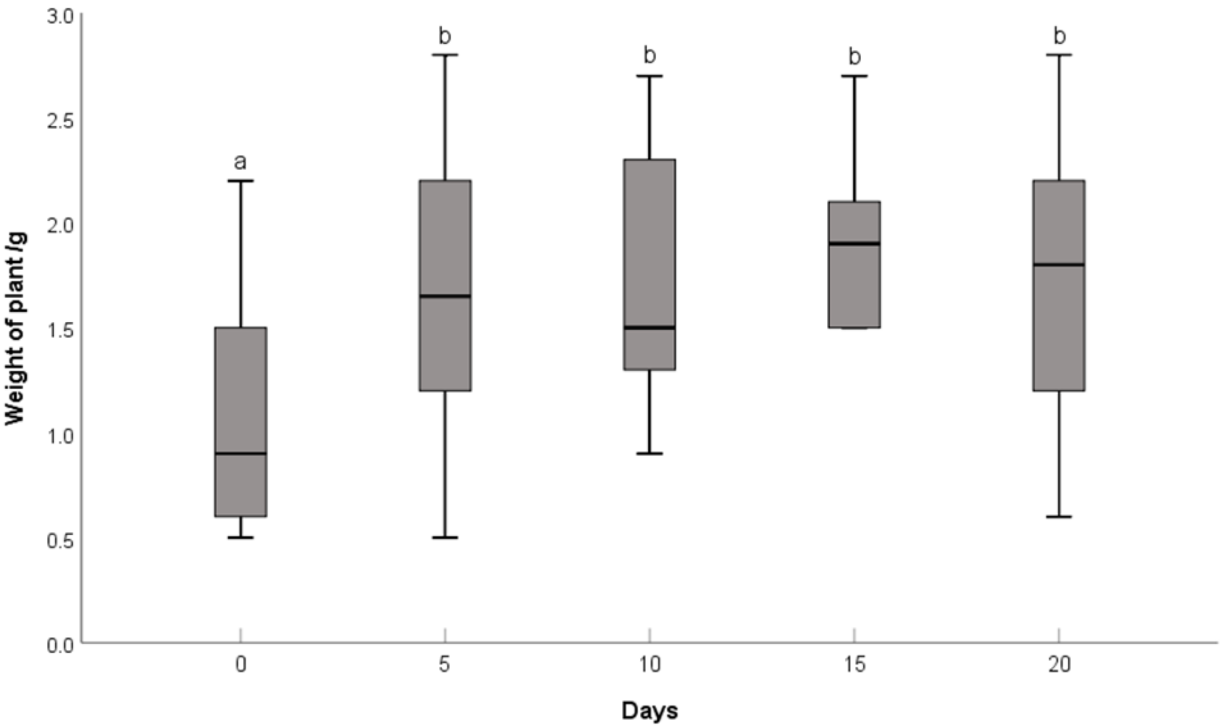

Supplementary Figure S1. Distribution and comparison of the weights of spinach plants used in pre-harvest simulation among days. Data are shown by integrated results ignoring different treatments to get more data on each day ($7 \leq n \leq 18$) due to the variable weights of triplicate samples for each treatment. Boxplots represent the median (horizontal line within the central box) with the first and third quartile, the whiskers show the minimum and maximum values. In the boxplot representation, 50% of data values are within the central box. Boxplots marked with different letters are statistically different ($p < 0.05$).
