# Supplementary material for: Behavior of the Biological Control Agent Bacillus thuringiensis subsp. aizawai ABTS-1857 and Salmonella enterica on Spinach Plants and Cut Leaves
Source: Front Microbiol. 2021 Feb 3;12:626029. doi: 10.3389/fmicb.2021.626029 (PMC7886684; doi:10.3389/fmicb.2021.626029)
Supplement: Supplementary file 2 [file Table_1.DOCX]

Supplementary Material

# Supplementary Tables

**Table S1.** Temperature and Humidity monitored during pre-harvest simulation in grow chamber (with a photoperiod of 12 h ascertained by a 250 W lamp) in April, 2019, Ghent, Belgium.

| Day | Temperature (°C ± SD ) | Temperature (°C ± SD) in period (five days before harvest) | Humidity (% RH ± SD) | Humidity (% RH ± SD) in period (five days before harvest) |
| --- | --- | --- | --- | --- |
| 0* | 20.7 ± 2.4 | 21.0 ± 0.2 | 51.8 ± 7.9 | 56.4 ± 4.3 |
| 1 | 20.9 ± 2.3 |  | 51.6 ± 4.3 |  |
| 2 | 21.3 ± 2.7 |  | 59.1 ± 6.3 |  |
| 3 | 21.1 ± 2.0 |  | 60.3 ± 5.5 |  |
| 4 | 21.1 ± 2.2 |  | 59.1 ± 5.0 |  |
| 5* | 22.1 ± 3.2 | 21.6 ± 1.0 | 48.3 ± 5.1 | 52.2 ± 10.0 |
| 6 | 22.8 ± 3.5 |  | 40.1 ± 5.1 |  |
| 7 | 21.5 ± 2.6 |  | 47.6 ± 10.0 |  |
| 8 | 21.0 ± 2.8 |  | 61.3 ± 2.7 |  |
| 9 | 20.3 ± 2.3 |  | 63.7 ± 2.2 |  |
| 10* | 21.8 ± 3.6 | 20.7 ± 0.7 | 64.6 ± 3.6 | 56.1 ± 6.6 |
| 11 | 21.0 ± 1.9 |  | 46.4 ± 3.4 |  |
| 12 | 19.9 ± 0.8 |  | 58.5 ± 1.8 |  |
| 13 | 20.5 ±1.0 |  | 56.3 ± 2.2 |  |
| 14 | 20.2 ± 0.9 |  | 54.6 ± 2.3 |  |
| 15* | 20.1 ± 1.0 | 21.2 ± 0.7 | 51.0 ± 4.4 | 49.9 ± 1.4 |
| 16 | 21.6 ± 0.4 |  | 47.9 ± 1.9 |  |
| 17 | 21.6 ± 0.9 |  | 49.3 ± 0.8 |  |
| 18 | 21.1 ± 1.0 |  | 51.3 ± 0.6 |  |
| 19 | 21.6 ± 0.2 |  | 49.9 ± 0.4 |  |
| 20* | 21.5 ± 0.3 |  | 51.3 ± 1.6 |  |

* The day harvested spinach plant at 8.00h-9.00h in the morning, on day 0 spinach plants were harvested after bacteria inoculation.
